# IL-27 Induced by Select *Candida* spp. via TLR7/NOD2 Signaling and IFN-β Production Inhibits Fungal Clearance

**DOI:** 10.4049/jimmunol.1501204

**Published:** 2016-06-03

**Authors:** Emmanuel C. Patin, Adam V. Jones, Aiysha Thompson, Mathew Clement, Chia-Te Liao, James S. Griffiths, Leah E. Wallace, Clare E. Bryant, Roland Lang, Philip Rosenstiel, Ian R. Humphreys, Philip R. Taylor, Gareth W. Jones, Selinda J. Orr

**Affiliations:** * Division of Infection and Immunity, Cardiff University School of Medicine, Cardiff CF14 4XN, United Kingdom;; † University Dental Hospital, Cardiff and Vale University Health Board, Cardiff CF14 4XY, United Kingdom;; ‡ Department of Veterinary Medicine, University of Cambridge, Cambridge CB3 0ES, United Kingdom;; § Institute of Clinical Microbiology, Immunology and Hygiene, University Hospital Erlangen, Friedrich Alexander University Erlangen-Nürnberg, 91054 Erlangen, Germany; and; ¶ Institute of Clinical Molecular Biology, Christian-Albrechts-University of Kiel, 24105 Kiel, Germany

## Abstract

*Candida* spp. elicit cytokine production downstream of various pathogen recognition receptors, including C-type lectin-like receptors, TLRs, and nucleotide oligomerization domain (NOD)–like receptors. IL-12 family members IL-12p70 and IL-23 are important for host immunity against *Candida* spp. In this article, we show that IL-27, another IL-12 family member, is produced by myeloid cells in response to selected *Candida* spp. We demonstrate a novel mechanism for *Candida parapsilosis*–mediated induction of IL-27 in a TLR7-, MyD88-, and NOD2-dependent manner. Our data revealed that IFN-β is induced by *C. parapsilosis,* which in turn signals through the IFN-α/β receptor and STAT1/2 to induce IL-27. Moreover, IL-27R (WSX-1)–deficient mice systemically infected with *C. parapsilosis* displayed enhanced pathogen clearance compared with wild-type mice. This was associated with increased levels of proinflammatory cytokines in the serum and increased IFN-γ and IL-17 responses in the spleens of IL-27R–deficient mice. Thus, our data define a novel link between *C. parapsilosis*, TLR7, NOD2, IFN-β, and IL-27, and we have identified an important role for IL-27 in the immune response against *C. parapsilosis*. Overall, these findings demonstrate an important mechanism for the suppression of protective immune responses during infection with *C. parapsilosis,* which has potential relevance for infections with other fungal pathogens.

## Introduction

Morbidity and mortality due to invasive fungal infections in hospitalized patients have increased in recent years, likely owing to medical advances resulting in more immunocompromised patients. *Candida* spp. are the most common cause of life-threatening invasive fungal infections in severely immunocompromised patients (1). From 2006 to 2007, *Candida* spp. were the fourth most common cause of health care–associated infections (2). An estimated 400,000 cases of life-threatening *Candida* infections occur per year, with mortality rates of 46–75%. Although several *Candida* spp. can cause disease, >95% of *Candida* infections are due to *C. albicans* (54%), *C. glabrata* (19%), *C. parapsilosis* (11%), and *C. tropicalis* (11%) (1, 3). In a direct comparison in mice, *C. albicans* and *C. tropicalis* were more pathogenic than *C. glabrata* and *C. parapsilosis* (4).

Colonization and development of candidiasis are determined by the interaction of *Candida* spp. with host immune cells. Myeloid cells such as monocytes, macrophages, and dendritic cells express pathogen recognition receptors that bind to the pathogen and initiate an immune response. Fungal cell wall components such as β-glucans and mannans are recognized by cell surface C-type lectin-like receptors, including Dectin-1, Dectin-2, and Mincle, and by TLRs such as TLR2 and TLR4 (5–9). More recently, it has been shown that nucleic acids and chitin from *Candida* spp. are sensed by the endosomal receptors TLR7 and TLR9 and the cytoplasmic receptor nucleotide oligomerization domain (NOD) 2 (10–12). Engagement of these receptors on myeloid cells results in the production of various cytokines, including TNF, IL-12, IL-23, IL-1β, IL-10, IL-6, and type I IFNs (IFN-α and IFN-β) (7, 10, 12–14). This activity in turn results in the induction of sustained Th1 and Th17 cell responses (6, 13, 15). Th1 cells produce IFN-γ, which has been shown to be critical in the control of candidiasis in mice (16, 17). In contrast, the protective role of Th17 cell–mediated cytokines, namely, IL-17 and IL-22, during infection with *Candida* spp. has been the subject of much debate. The Th17 cell response was reported to protect against disseminated, oropharyngeal, and mucocutaneous models of candidiasis (18–20) while increasing disease and susceptibility in a gastrointestinal model (21).

Dectin-1–, Dectin-2–, and Mincle-deficient mice display reduced myeloid/innate-derived cytokine/chemokine production and increased susceptibility to *Candida* infections (7, 13, 14). Consequently, Th1 and Th17 cell responses are severely attenuated with Dectin-2 blockade and Dectin-1 deficiency (6). The function of TLRs in in vivo models of candidiasis has been extensively studied, and mice lacking TLR2, TLR4, or TLR9 demonstrate varying levels of susceptibility to fungal infections, depending on the fungal species and the route of infection (22). Of interest, TLR7-deficient mice display increased susceptibility to low-dose systemic *C. albicans* infection; however, no differences were observed in susceptibility of TLR7 null mice to higher doses of *C. albicans* when compared with their wild-type (WT) counterparts (23). To our knowledge, no study has been reported on the role of NOD2 in the host defense to *Candida* spp. in mice. However, preliminary investigations in humans suggested no significant involvement of NOD2 in the recognition of *C. albicans* (24).

IL-12 family members (IL-12p70, IL-23, IL-27, and IL-35) are important regulators of T cell responses. Despite the structural similarities brought about through the sharing of common α and β subunit chains, this heterodimeric cytokine family has greatly differential effects on T cells (25). IL-12 and IL-23 are predominantly considered proinflammatory. IL-12 supports Th1 cell differentiation, whereas IL-23 enhances Th17 activities (25). In contrast, IL-35 is derived from regulatory T cells and suppresses effector T cell responses (26). IL-27 is a potent T cell immunomodulator that has both pro- and anti-inflammatory properties (27). IL-27 negatively regulates IL-2 signaling to inhibit effector T cell responses and limit host disease (28, 29). However, early studies suggested a proinflammatory role through enhancement of early Th1 cell differentiation (30). IL-27 also potently inhibits the differentiation of Th17 cells to protect against Th17-associated disease (31, 32). In agreement with this, patients with gain-of-function STAT1 mutations display enhanced responses to IL-27, IFN-γ, and IFN-α, and they demonstrate reduced IL-17 responses (33–35). In addition, IL-27 inhibits the development of inducible T regulatory cells (Tregs) (36), whereas other studies show that IL-27 promotes the growth and survival of Tregs at local sites of infection (37, 38).

IL-27 is a heterodimeric cytokine that is mainly produced by APCs [monocytes, macrophages, and dendritic cells (DCs)]. IL-27 consists of the p28 and EBI-3 (Epstein–Barr virus–induced gene 3) chains and it signals through the unique IL-27R subunit paired with gp130 (39). IL-27 is induced by TLR signaling via MyD88 and NF-κB (40) and via MyD88-independent Trif/IRF3 signaling (41). In addition, type I and type II IFNs induce IL-27 via the activation of several IRFs (IRF1, IRF3, IRF7, and IRF9) (41–44). TNF has also been shown to induce IL-27 (45). Of note, infections with various pathogenic agents, including *Mycobacterium tuberculosis* and *Toxoplasma gondii*, have been associated with increased expression of IL-27 (28, 29). However *Aspergillus fumigatus*–infected DCs did not produce significant amounts of IL-27 (46). Heat-killed *C. albicans* has been shown to enhance LPS-induced IL-27 production (47); however, it is currently unknown whether any *Candida* spp. directly induce IL-27 production and whether IL-27 is important for antifungal immunity.

In this article, we show for the first time, to our knowledge, that some *Candida* spp. induce IL-27 production in myeloid cells, whereas *C. albicans* does not. We show that *C. parapsilosis*–induced IL-27 is dependent on phagocytosis, TLR7/MyD88, and NOD2, and the resultant production of IFN-β, which signals through IFN-α/β receptor (IFNAR) and STAT1/2 to induce IL-27. Importantly, IL-27R–deficient mice displayed enhanced clearance of systemic infection with *C. parapsilosis*. This was associated with increased levels of proinflammatory cytokines in the serum and increased IFN-γ and IL-17 responses in the spleens of IL-27R–deficient mice. Thus, IL-27 plays an important role in immune response and pathogen clearance during infection with *C*. *parapsilosis.*

## Materials and Methods

### Mice

*Il27ra^−/−^* (48), *Clec7a^−/−^, Tlr2^−/−^, Tlr4^−/−^, Tlr2/4^−/−^, Card9^−/−^, Il10^−/−^*, and age- and gender matched control C57BL/6 mice were maintained and handled according to institutional and U.K. Home Office guidelines. *Clec4e^−/−^* and *Fcre1g^−/−^* mice were maintained at the University Hospital Erlangen. *Nod2^−/−^* mice were maintained according to German and European Union guidelines.

### Ethics statement

This study was performed in strict accordance with the Project License and procedures that were approved by Cardiff University Animal Welfare and Ethical Review Body and the U.K. Home Office. The animal care and use protocol adhered to the Animals (Scientific Procedures) Act 1986.

### Reagents

Ultrapure LPS, Pam_3_Csk_4_, and TDB were purchased from Invivogen. Curdlan (Wako Chemicals), a β-1,3-glucan preparation from *Alcaligenes faecalis,* was used in this study. GM-CSF, M-CSF, and TNF were purchased from PeproTech. Cytochalasin D (Sigma-Aldrich) and the TNF antagonist etanercept, a soluble TNFR:Fc fusion protein (Wyeth Europa) were used in this study. IFN-β, and ELISAs for IFN-β and IFN-α, were purchased from R&D. Anti-Ly6C, anti-CD11b, and anti-B220 were purchased from BioLegend. Anti–IL-27p28 was purchased from BD Biosciences. Anti-phosphoSTAT1, anti-STAT1, anti-IRF1, and anti-IRF3 were purchased from Cell Signaling Technology. Anti-IRF7 and anti–Lamin B1 (Abcam) were used in this study. Anti-NOD2 (clone H-300) and anti-TLR7 (clone V-20) were from Santa Cruz Biotechnology, and secondary Abs for immunofluorescence (Cy5 donkey anti-rat and Cy2 donkey anti-rabbit) were from Jackson ImmunoResearch. DAPI nuclei stain was from Life Technologies. Rhodamine Green-X was purchased from Invitrogen. *C. albicans* SC5314 from American Type Culture Collection was used in this study. *C. albicans* strains JIMS500019 (vaginal isolate), J981318 (vaginal isolate), and AM2005/0463 (blood isolate), *C. glabrata* strains SCS74761 and SCSB5311, *C. tropicalis* strains AM2007/0112 and SCS74663, and *C. parapsilosis* strains AM2005/0207 and SCSB5882 were a kind gift from Dr. Donna MacCallum (University of Aberdeen, Aberdeen, Scotland).

### Preparation of *Candida* cultures

*Candida* spp. were plated on yeast extract/peptone/dextrose (YPD) agar, cultured for 20 h in YPD broth, washed three times with PBS, and resuspended at the required concentration in PBS. *Candida* spp. were heat killed by boiling for 30 min at 100°C.

### Cell culture

Bone marrow cells were flushed out of the femurs and tibias of mice. Bone marrow–derived macrophages (BMDMs) were generated by culturing cells for 6–7 d in DMEM medium containing 10% FBS, 5% horse serum, 2 mM l-glutamine, penicillin/streptomycin, HEPES, and 10 ng/ml M-CSF. BM-derived DCs (BMDCs) were generated by culturing cells for 8 d in RPMI 1640 medium containing 10% FBS, 2 mM l-glutamine, penicillin/streptomycin, HEPES, NEAA, sodium pyruvate, 2-ME, and 10 ng/ml GM-CSF.

### Cell stimulations

BMDMs were harvested, resuspended in RPMI 1640 and 10% FBS, plated at 1 × 10^7^ cells/10 cm^2^, and then left overnight at 37°C. Media were removed, and 1 × 10^7^ *Candida* CFU were added per plate for the indicated times. Amphotericin B (Fungizone) (2.5 μg/ml) was added 2 h after stimulation. Cytosol and nuclear extracts were prepared using Novagen NucBuster Protein Extraction Kit (Merck Millipore). Lysates were clarified by centrifugation, and nonreducing sample buffer was added to lysates and heated for 5 min at 95°C. Lysates were separated by SDS-PAGE (Bio-Rad), transferred to polyvinylidene difluoride membrane (Merck Millipore), and analyzed by Western blot.

### Cytokine assays

BMDMs and BMDCs were plated at a density of 1 × 10^5^ cells per well in a 96-well plate in RMPI 1640 containing 10% FBS. BMDMs and BMDCs were stimulated with *Candida* spp. yeast, heat-killed yeast, LPS, Pam_3_Csk_4_, curdlan, TDB, or IFN-β for 24 h. Inhibitors or blocking Abs were added to cells 30 min to 1 h prior to stimulation. Amphotericin B (Fungizone) was added 2 h after stimulation for all experiments except for the cytochalasin D experiment, in which amphotericin B (Fungizone) was added 4 h after stimulation. Cell culture supernatants were recovered and assayed for cytokine by ELISA (Affymetrix eBioscience), according to the manufacturer’s protocol. The IL-27 ELISA detects the heterodimer (IL-27p28 and EBI3).

### RNA, cDNA, and real-time quantitative PCR

RNA was extracted using TRIzol (Life Technologies) and further purified using the RNeasy Mini Kit with on-column DNase treatment (QIAGEN). cDNA was synthesized from total RNA using the TaqMan Reverse Transcription Kit (Invitrogen). Gene expression was determined on the QuantStudio 12K Flex Real-Time PCR System (Life Technologies) using ABI TaqMan Primer and Probe Sets (Life Technologies). Gene expression was normalized against *Hprt1*.

### In vivo *Candida* spp. infections

Mice were matched by gender and age (8–12 wk old), and 100 μl *C. parapsilosis* or *C. albicans* in PBS was injected i.v. Mice were monitored and weighed daily. Experiments were continued for ≤42 d for *C. parapsilosis* or ≤30 d for *C. albicans*. For both in vivo models, mice were bled by cardiac puncture after sacrifice, and kidneys, brains, and spleens were harvested. The left kidney was placed in PBS and homogenized, and serial dilutions were plated on YPD agar containing 50 μg/ml chloramphenicol. The plates were cultured for 48 h, and CFU were calculated per gram of organ. For the *C. parapsilosis* model, the right kidney was placed in 10% formalin, embedded in paraffin wax blocks, processed using an automated tissue processor, sectioned at 4 μm, deparaffinized, and stained with H&E and periodic acid–Schiff according to standard protocols. The spleens were homogenized, RBCs were lysed with ACK lysis buffer, and the cells were washed with PBS. The cells were resuspended in IMDM containing 10% FBS, 2 mM l-glutamine, penicillin/streptomycin, and 2-ME; plated; and restimulated with *C. parapsilosis* for 48 h or with PMA (50 ng/ml) and ionomycin (0.5 μg/ml) in the presence of 0.2% brefeldin A for 4 h. IFN-γ and IL-17 levels were measured by ELISA, or IFN-γ– and IL-17A–producing cells were analyzed by flow cytometry.

### Histology scoring

Kidneys were bisected and examined at 4 μm, and the cortex and medulla were individually assessed for the presence of neutrophils (acute inflammation) and lymphocytes/plasma cells (chronic inflammation) and scored as follows: score 0 = no inflammation; score 1 = <3 foci of inflammation; score 2 = 4–6 foci of inflammation; score 3 = >6 foci of inflammation, but less than 25% of the kidney affected; score 4 = >25% of the kidney affected. Each individual focus was subsequently assessed to determine the area of inflammation (in square micrometers) using cellSens Software (Olympus Corporation, Tokyo, Japan), and the total percentage of the affected kidney was determined using the cumulative area of inflammation/total area of the kidney section. Scoring was performed by a pathologist blinded to the experimental groups.

### Flow cytometry

Bone marrow or spleen cells from naive mice were stimulated with *Candida* spp. for 22 h in the presence of brefeldin A for the last 12 h. IL-27p28–producing cells were determined by flow cytometry.

### Immunofluorescence

Intracellular localization of NOD2 and TLR7 was assessed by immunofluorescence, as described previously (12). Briefly, BMDMs were harvested, resuspended in RPMI 1640 supplemented with 10% FBS, plated into 24-well plates containing coverslips at a density of 1 × 10^5^ per well, and cultured overnight. Media were removed from BMDMs, and 1 × 10^5^ *Candida* spp. in fresh media was added to BMDMs for 1 h at 37°C. Cells were fixed with 4% paraformaldehyde in PBS for 30 min and permeabilized with 0.5% Triton-X 100 in PBS for 10 min. Cells were blocked with 5% donkey serum in PBS for 30 min prior to staining with anti-NOD2 or anti-TLR7 for 1 h. Cells were then incubated with Cy5 anti-rat and Cy2 anti-rabbit for 1 h, and coverslips were mounted onto glass microscopic slides using mounting medium containing DAPI. Immunofluorescence staining was visualized using a Zeiss Apotome microscope fitted with a ×63 oil immersion lens. For the *Candida* counts, 3.2 × 10^8^ *Candida* CFU were incubated with Rhodamine Green-X for 30 min and washed extensively with PBS to remove any unbound Rhodamine Green-X before use. Media were removed from BMDMs, and 1 × 10^5^ Rhodamine Green-X–labeled *Candida* spp. in fresh media was added to BMDMs for 1 h at 37°C before fixing with 4% paraformaldehyde and mounting coverslips onto glass microscopic slides using mounting medium containing DAPI. Images were taken at ×20 magnification from five different slide sections per experiment, and the number of *Candida* cells per infected macrophage was counted.

### Statistical methods

Data are presented as means ± SEM and are representative of two to four independent experiments. One-way ANOVA followed by Bonferroni’s posttest was used for statistical analysis when multiple groups were analyzed. Student *t* test or Mann–Whitney *U* test was used for statistical analysis when two groups were analyzed. When data did not follow a Gaussian distribution, it was transformed by Y = sqrt(Y + 0.5) (49) and analyzed by Student *t* test. Statistical significance was set at *p < 0.05, **p < 0.005, and ***p < 0.001.

## Results

### IL-27 production is induced by *C. parapsilosis* in myeloid cells

**FIGURE 1.**
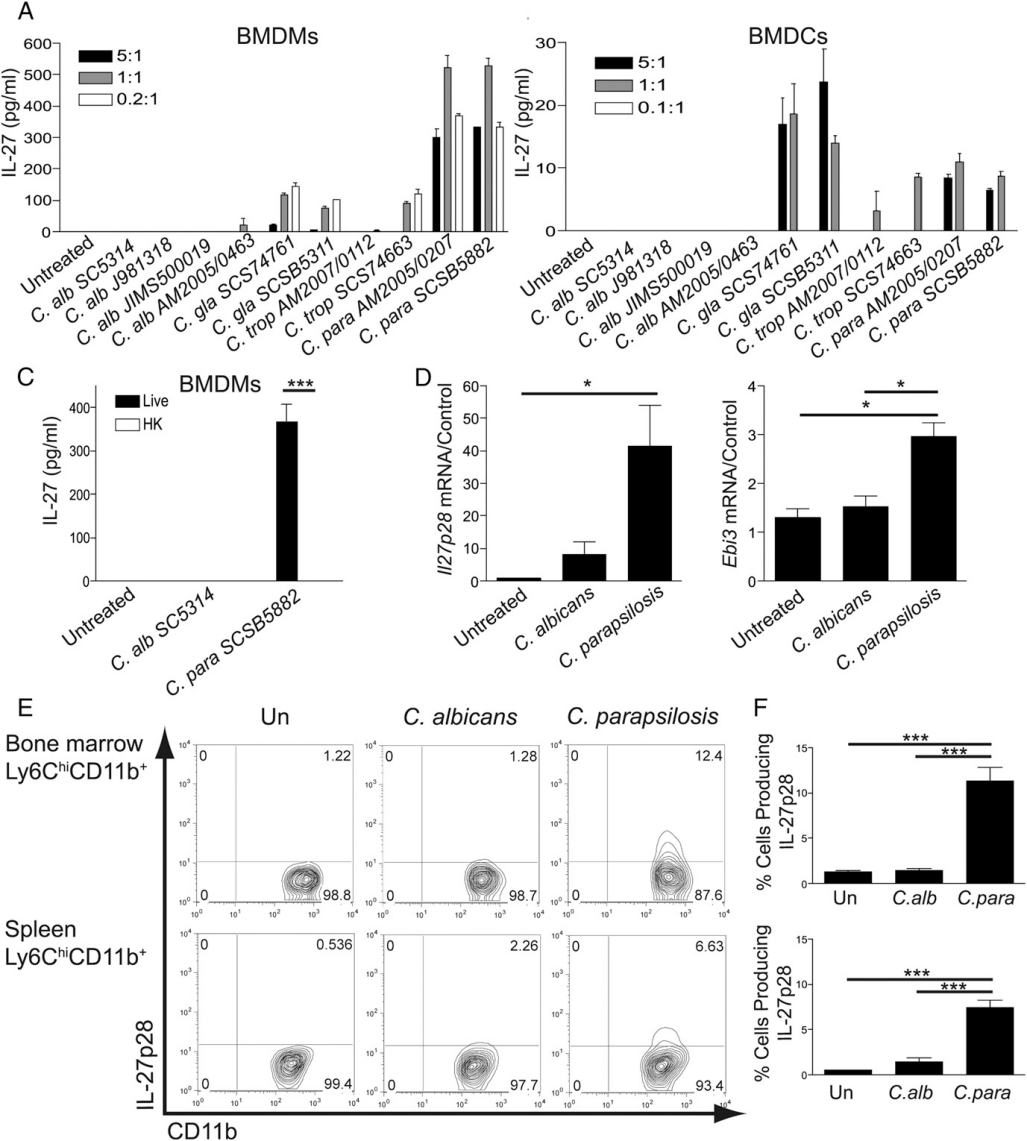
**IL-27** Production is induced by *C*. *parapsilosis* in myeloid cells. BMDMs (**A**) and BMDCs (**B**) from WT mice were stimulated with 10 species and strains of *Candida* at the indicated ratios of *Candida*/cells. Cytokine levels in the supernatants were measured after 24-h incubation. (**C**) BMDMs from WT mice were stimulated with live or heat-killed *Candida* spp. Cytokine levels in the supernatants were measured after 24-h incubation. (A–C) Results are presented as means ± SEM of three replicates, and data are representative of three independent experiments. ***p < 0.001. (**D**) BMDMs were stimulated with *Candida* spp. for 24 h. RNA was isolated, cDNA was prepared, and *Il27p28* and *Ebi3* mRNA transcripts were detected by real-time quantitative PCR. mRNA levels were normalized to *Hprt1*. Graph displays mean ± SEM of four biological replicates from three independent experiments. *p < 0.05. (**E** and **F**) Splenic and BM cells were cultured with *Candida* spp. at a ratio of 1:1 *Candida*/cells for 22 h. IL-27p28 levels were analyzed by flow cytometry. Flow cytometry plots were gated on singlet, autofluorescent^−^, B220^−^, and Ly6C^hi^CD11b^+^ cells. Data are representative of three independent experiments. (F) Graphs display mean ± SEM percentage of cells expressing IL-27p28 from three mice analyzed by flow cytometry. Graphs are representative of three independent experiments. **p < 0.005 (one-way ANOVA, Bonferroni’s posttest).

As various *Candida* spp. induce IL-12 and IL-23 and both of these IL-12 family members are involved in the antifungal immune response (17, 18, 21), we postulated that an additional IL-12 family member, IL-27, may play an important role in the antifungal response to *Candida*. We first aimed to determine whether any *Candida* spp. induce IL-27 production. To investigate this possibility, we stimulated BMDMs and BMDCs with two to four strains each of *C. albicans, C. glabrata, C. tropicalis*, and *C. parapsilosis*. Of interest, although all four strains of *C. albicans* failed to induce production of IL-27, *C. parapsilosis, C. glabrata*, and one strain of *C. tropicalis* induced varying levels of IL-27 (Fig. 1A, 1B). BMDCs produced very low levels of IL-27, and these were considerably lower than those produced by BMDMs. As *C. parapsilosis* induced the highest level of IL-27, the following experiments focused on the commonly used laboratory strain of *C. albicans* (SC5314) versus a clinically isolated strain of *C. parapsilosis* (SCSB5882). Of note, heat-killed *C. parapsilosis* failed to elicit IL-27 production (Fig. 1C), indicating that live *C. parapsilosis* is necessary to induce robust IL-27 levels. Both subunits of IL-27 (*Il27p28* and *Ebi3*) were induced at the RNA level in response to *C. parapsilosis* and, to a much lesser extent, by *C. albicans* (Fig. 1D). As BMDMs and BMDCs are differentiated in vitro, we wanted to confirm whether *C. parapsilosis* could induce IL-27 in primary cells stimulated ex vivo. To this end, bone marrow and splenic cells were stimulated with *C. albicans* or *C. parapsilosis* for 22 h. As with the BMDMs and BMDCs, we observed that *C. parapsilosis* induced IL-27p28 in both bone marrow and splenic B220^−^Ly6C^hi^CD11b^+^ cells, whereas *C. albicans* did not (Fig. 1E, 1F). These data demonstrate interstrain/interspecies variability in the ability of *Candida* to induce IL-27 in myeloid cells.

### *C. parapsilosis*–induced IL-27 production is dependent on phagocytosis, MyD88, TLR7, and NOD2

**FIGURE 2.**
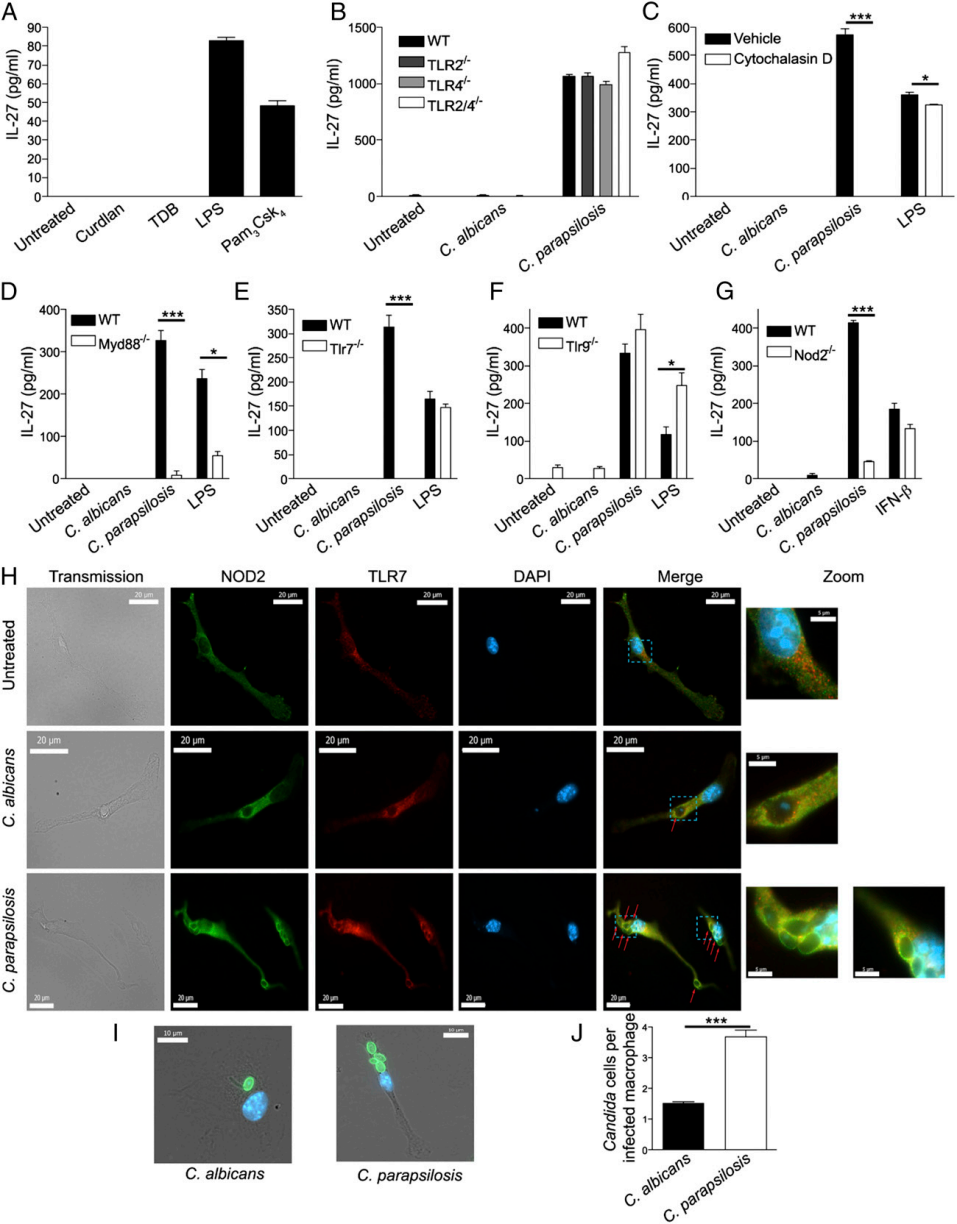
*Candida-*induced IL-27 production is dependent on phagocytosis, MyD88, TLR7, and NOD2. (**A**) BMDMs from WT mice were stimulated with 10 μg/ml curdlan, 10 μg/ml TDB, 10 ng/ml LPS, and 1 μg/ml Pam_3_Csk_4_. Cytokine levels in the supernatants were measured after 24-h incubation. (**B**) BMDMs from WT, *Tlr2^−/−^*, *Tlr4^−/−^*, and *Tlr2/4^−/−^* mice were stimulated with *Candida* spp. Cytokine levels in the supernatants were measured after 24-h incubation. (**C**) BMDMs from WT mice were stimulated with *Candida* spp. in the presence of vehicle control or 10 μM cytochalasin D. Cytokine levels in the supernatants were measured after 24-h incubation. *p < 0.05, ***p < 0.001. (**D**–**G**) BMDMs from WT and *Myd88^−/−^* mice (D), WT and *Tlr7^−/−^* mice (E), WT and *Tlr9^−/−^* mice (F), and WT and *Nod2^−/−^* mice (G) were stimulated with *Candida* spp. and 200 ng/ml LPS or 200 U/ml IFN-β. Cytokine levels in the supernatants were measured after 24-h incubation. For all graphical data, results are presented as means ± SEM of three replicates, and data are representative of two to four independent experiments. *p < 0.05 (D and F), ***p < 0.001 (D, E, and G) (one-way ANOVA, Bonferroni’s posttest). (**H**) BMDMs from WT mice were unstimulated or stimulated with *C. albicans* or *C. parapsilosis* (red arrows) for 1 h. Cells were stained with anti-NOD2 (green), anti-TLR7 (red), and DAPI (blue). Scale bars, 20 μm. Images on the far right are zoomed sections (blue dashed boxes) from the merged images; scale bars, 5 μm. Data are representative of two to four independent experiments. (**I** and **J**) BMDMs from WT mice were stimulated with Rhodamine Green-X–labeled *C. albicans* or *C. parapsilosis* (green) for 1 h. Cells were stained with DAPI (blue). (I) Images displayed are overlaid on transmission images. Scale bars, 10 μm. Data are representative of three independent experiments. (J) Graph displays mean number of *Candida* cells per infected BMDM. Data are representative of three independent experiments. ***p < 0.001 (Mann–Whitney *U* test).

TLR ligands have been shown to induce IL-27 (40, 41). Given that *C. parapsilosis* induced robust IL-27 production in BMDMs, we next sought to determine which receptors were involved in this response. BMDMs were stimulated with a Dectin-1 ligand (curdlan), a Mincle ligand (TDB), and ligands for TLR4 (LPS) and TLR2 (Pam_3_Csk_4_). Only the TLR2 and TLR4 ligands induced IL-27 (Fig. 2A). As *Candida* spp. contain TLR2/4 ligands in their cell wall, we hypothesized that the IL-27 production was TLR2/4 dependent. Surprisingly, *C. parapsilosis*–induced IL-27 production was TLR2/4 independent (Fig. 2B). We therefore investigated whether some C-type lectin-like receptors and their associated signaling pathways were required for IL-27 secretion. In agreement with the ligand data from Fig. 2A, BMDMs from Dectin-1 (*Clec7a*) null mice (Supplemental Fig. 1A) and Mincle (*Clec4e*) null mice (Supplemental Fig. 1B) confirmed that these receptors were not required for IL-27 induction. As both Mincle and Dectin-2 signal through the FcεRIγ adaptor and several fungal-associated receptors signal through CARD9, we also stimulated FcεRIγ and CARD9 null BMDMs with *Candida* spp. to find that these signaling components were not required for IL-27 production (Supplemental Fig. 1C, 1D). As none of the expected cell surface receptors were responsible for *C. parapsilosis*–induced IL-27 production, we next investigated whether phagocytosis was involved. To this end, BMDMs were incubated with cytochalasin D. This resulted in ablation of *C. parapsilosis*–induced IL-27 production, whereas the effect on LPS was much less pronounced (Fig. 2C). This finding suggested the involvement of endosomal/intracellular receptors. Biondo et al. (23, 50) demonstrated that fungal RNA and fungal DNA signal through the endosomal receptors TLR7 and TLR9, respectively. In addition, NOD2 has recently been shown to respond to the fungal ligand chitin in collaboration with TLR9 (12), and NOD2 responds to viral RNA (51). In line with these data, we next examined whether TLR7/9-MyD88 or NOD2 was involved in mediating *C. parapsilosis*–induced IL-27 production. Stimulation of MyD88 (Fig. 2D) and TLR7 (Fig. 2E) null BMDMs demonstrated almost complete ablation of *C. parapsilosis*–induced IL-27 production. However, *C. parapsilosis*–induced IL-27 secretion was independent of TLR9 (Fig. 2F). Of interest, NOD2 (Fig. 2G) null cells showed a significant reduction in IL-27 production. These data indicate that *C. parapsilosis*–induced IL-27 production is dependent on phagocytosis, TLR7/MyD88, and NOD2.

As Wagener et al. (12) observed that TLR9 and NOD2 colocalized in response to the fungal ligand chitin, we aimed to determine whether TLR7 and NOD2 colocalized in a similar manner in response to *C. parapsilosis*. To this end, WT BMDMs were left unstimulated or stimulated with *C. albicans* or *C. parapsilosis* for 1 h. Although some colocalization of TLR7 and NOD2 was observed in unstimulated cells, colocalization of TLR7 and NOD2 increased in response to *Candida* spp. (Fig. 2H). Colocalization of TLR7 and NOD2 surrounding yeast cells was particularly evident in *C. parapsilosis*–treated BMDMs. Of note, *Candida*-infected BMDMs appeared to contain more *C. parapsilosis* cells than *C. albicans* cells. To confirm this observation, *C. albicans* and *C. parapsilosis* were labeled with Rhodamine Green-X, WT BMDMs were stimulated with the labeled *Candida* spp. for 1 h, and *Candida* cells per infected BMDM were counted. In agreement with previous findings (52), we observed that on a per cell basis, infected BMDMs ingested more *C. parapsilosis* cells than *C. albicans* cells (Fig. 2I, 2J). These data indicate that TLR7 and NOD2 colocalize in response to *Candida* spp. in a manner similar to TLR9 and NOD2 colocalization.

### IL-27 is a late-induced protein

**FIGURE 3.**
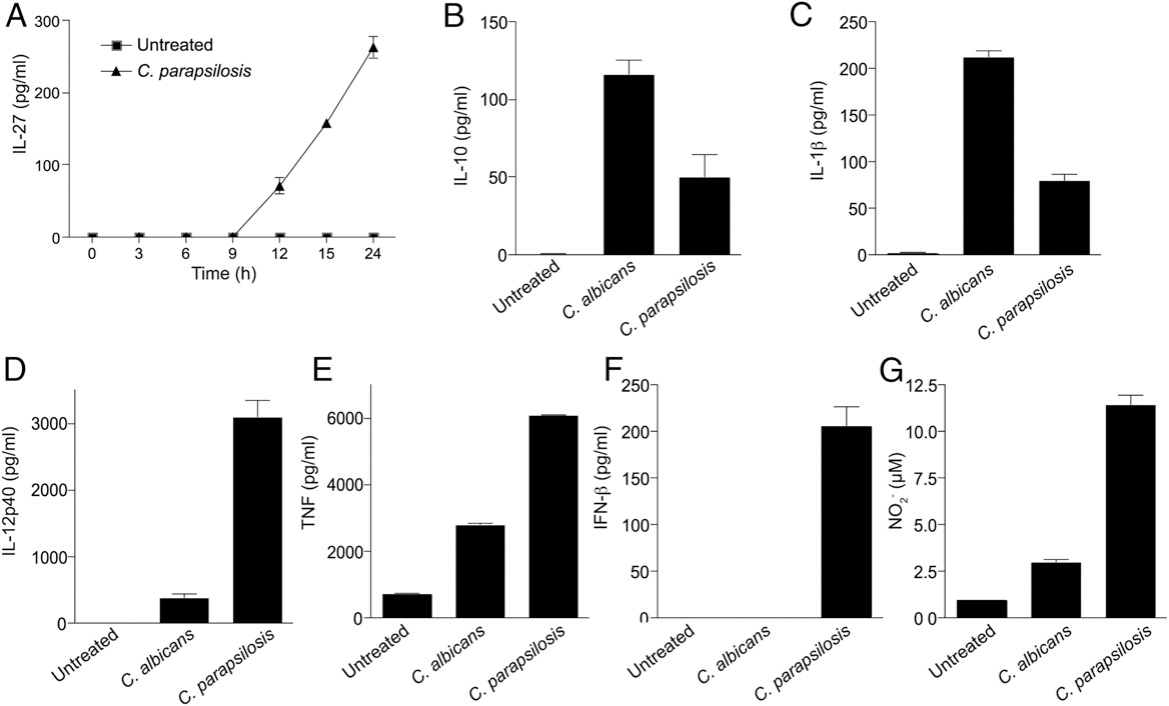
IL-27 is a late-induced protein. (**A**) BMDMs from WT mice were stimulated with *C. parapsilosis*, and IL-27 levels in the supernatants were measured after the indicated timepoints. (**B**–**F**) BMDMs from WT mice were stimulated with *Candida* spp., and cytokine levels in the supernatants were measured after 24-h incubation. (**G**) BMDMs from WT mice were stimulated with *Candida* spp., and NO_2_^−^ levels in the supernatants were measured after 24-h incubation. For all graphical data, results are presented as means ± SEM of three replicates and data are representative of three to four independent experiments.

We next sought to examine the kinetics of IL-27 production to determine whether it is directly induced by *C. parapsilosis* or whether an intermediate is involved. Interestingly, IL-27 production was only detected after 9- to 12-h stimulation with *C. parapsilosis* (Fig. 3A). As this is a late-induced protein, this suggested that an intermediate is involved in the induction of IL-27 by *C. parapsilosis*. To identify potential intermediates, we examined the expression profile of various other cytokines in response to the two *Candida* spp. IL-27 promotes the induction of IL-10 (53–55), and IL-10 has recently been shown to inhibit MyD88-dependent IL-27 release in macrophages (56), suggesting that IL-10 would not likely be responsible for the induction of IL-27 in our experiments. In agreement with this, IL-10 showed a differential expression profile to IL-27 in response to *Candida* spp. (Fig. 3B). IL-1β also showed a differential expression profile to IL-27 in response to *Candida* spp. (Fig. 3C). In addition, induction of IFN-α was absent in response to *Candida* spp. despite significant induction of *Ifna4* at the RNA level (data not shown). In contrast, IL-12p40, TNF, and, in particular, IFN-β showed similar response patterns to IL-27 (Fig. 3D–F). Notably, TNF has previously been shown to be an early-induced gene in response to β-glucan, whereas IL-10 and IL-12p40 were late-induced genes (57). Both TNF and IFN-β have previously been shown to induce IL-27 (43, 45). We therefore postulated that TNF and IFN-β may function as intermediates in *C. parapsilosis*–induced IL-27 production. In support of a role for IFN-β, inducible NO synthase is an IFN-β–inducible gene and NO_2_^−^ production displayed a similar response to that of IL-27 (Fig. 3G).

### *C. parapsilosis*–induced IL-27 production is IFN-β dependent

**FIGURE 4.**
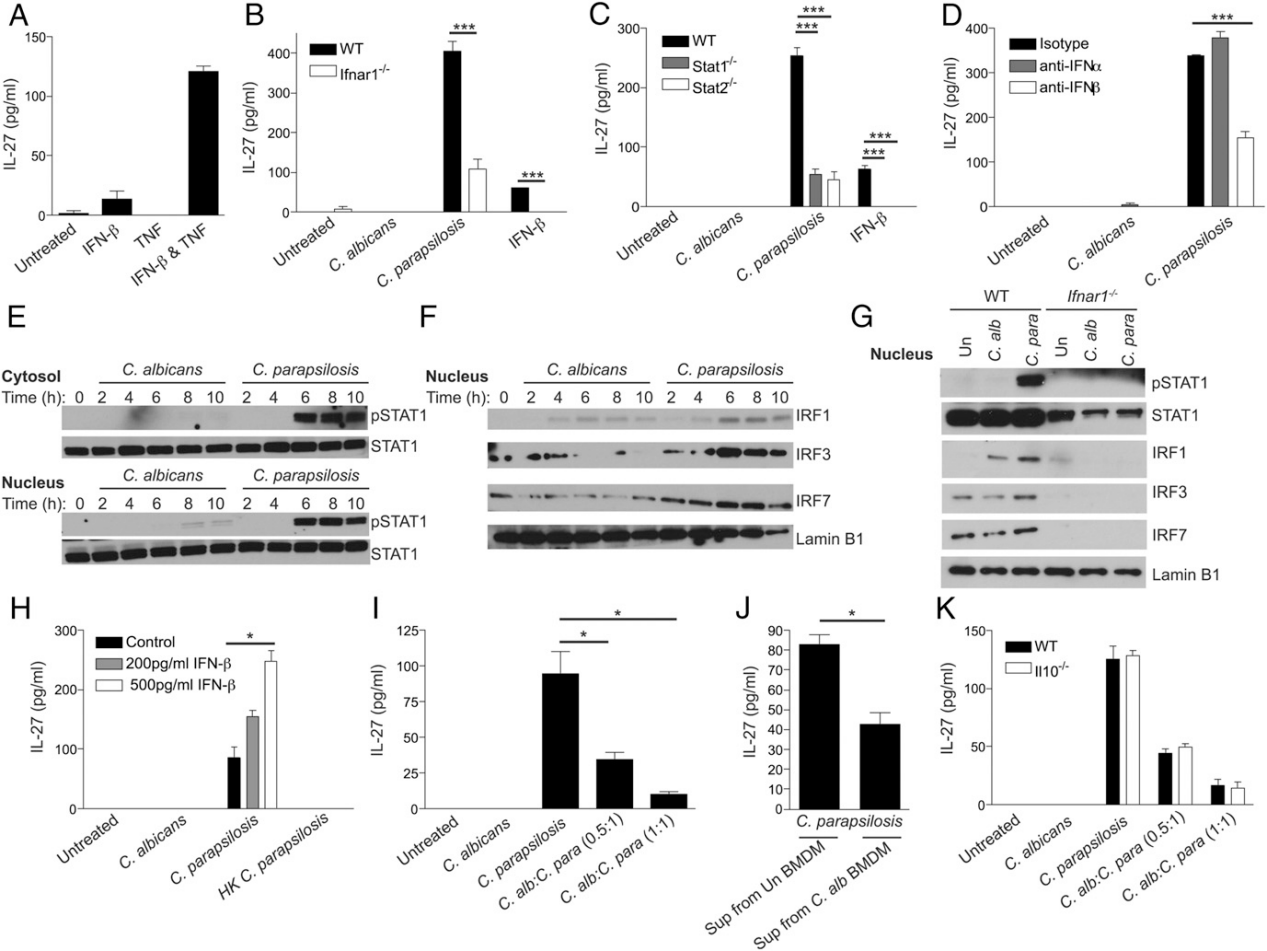
*Candida-*induced IL-27 production is IFN-β dependent. (**A**) BMDMs from WT mice were stimulated with IFN-β (200 U/ml) or TNF (10 ng/ml), and cytokine levels in the supernatants were measured after 24-h incubation. (**B** and **C**) BMDMs from WT and *Ifnar1^−/−^* mice (B) and WT, *Stat1^−/−^*, and *Stat2^−/−^* mice (C) were stimulated with *Candida* spp., and cytokine levels in the supernatants were measured after 24-h incubation. ***p < 0.001 (one-way ANOVA, Bonferroni’s posttest). (**D**) BMDMs from WT mice were stimulated with *Candida* spp. in the presence of isotype control or blocking Abs to IFN-α or IFN-β (10 μg/ml). Cytokine levels in the supernatants were measured after 24-h incubation. For all graphical data, results are presented as means ± SEM of three replicates and data are representative of three to four independent experiments. ***p < 0.001 (one-way ANOVA, Bonferroni’s posttest). (**E**–**G**) BMDMs from WT mice (E and F) or WT and *Ifnar1^−/−^* mice (G) were stimulated for the indicated times (E and F) or 6 h (G) with 1:1 *Candida*/cells. (E) Cytosolic and nuclear fractions were immunoblotted with anti-pSTAT1 and anti–STAT-1. (F) Nuclear fractions were immunoblotted with anti-IRF1, anti-IRF3, anti-IRF7, and anti-Lamin B1. (G) Nuclear fractions were immunoblotted with anti-pSTAT1, anti-STAT1, anti-IRF1, anti-IRF3, anti-IRF7, and anti–Lamin B1. (E–G) Data are representative of two independent experiments. (**H**) BMDMs from WT mice were stimulated with live or heat-killed *Candida* spp. in the presence or absence of IFN-β, and Il-27 levels in the supernatants were measured after 24-h incubation. (**I**) BMDMs from WT mice were stimulated with *C. albicans, C. parapsilosis*, or both spp., and IL-27 levels in the supernatants were measured after 24-h incubation. (**J**) BMDMs from WT mice were unstimulated or stimulated with *C. albicans* for 24 h. The supernatants from these cells were added to WT BMDMs stimulated with *C. parapsilosis*, and IL-27 levels in the supernatants were measured after 24-h incubation. (**K**) BMDMs from WT and *Il10^−/−^* mice were stimulated with *C. albicans, C. parapsilosis*, or both spp., and IL-27 levels in the supernatants were measured after 24-h incubation. (H–K) For all graphical data, results are presented as means ± SEM of three replicates, and data are representative of two to four independent experiments. *p < 0.05 [one-way ANOVA, Bonferroni’s posttest (H and I), Student *t* test (J)].

In agreement with previous findings, we observed that IFN-β can induce IL-27 in BMDMs (Fig. 4A); however, we saw no IL-27 production in response to TNF at the concentration used (10 ng/ml). Of interest, though, IFN-β and TNF synergistically induced robust IL-27 production (Fig. 4A). To dissect the contribution of TNF to *C. parapsilosis*–induced IL-27 production, WT BMDMs were stimulated with *Candida* spp. in the presence of the TNF inhibitor etanercept. This showed only a minor effect on IL-27 production (Supplemental Fig. 1E), despite a significant reduction in TNF levels (Supplemental Fig. 1F) in the presence of etanercept. We next evaluated IFN-β as the key intermediate in *C. parapsilosis*–induced IL-27 production. When IFNAR1 (Fig 4B) and STAT1/2 (Fig. 4C) null BMDMs were stimulated with *Candida* spp., we observed that the IFN pathway was critical in driving the IL-27 response. Furthermore, when BMDMs were stimulated with *C. parapsilosis* in the presence of blocking Abs to IFN-α and IFN-β, a substantial reduction in IL-27 was detected only in the presence of the anti–IFN-β Ab (Fig. 4D).

We have demonstrated differential induction of IL-27 in response to *C. parapsilosis* versus *C. albicans* (see Fig. 1A). To further determine whether this differential control of IL-27 was linked to the IFN response, we investigated downstream signaling pathways. In this context, the robust IL-27 response to *C. parapsilosis* was associated with strong and prolonged phosphorylation of STAT1 in both the cytosol and the nucleus (Fig. 4E) and with translocation of IRF1, IRF3, and IRF7 to the nucleus (Fig. 4F). Conversely, the absence of an IL-27 response to *C. albicans* was associated with weak and transient phosphorylation of STAT1 and translocation of IRF1, IRF3, and IRF7 (Fig. 4E, 4F). In agreement with these data, STAT1 phosphorylation and translocation of IRF1, IRF3, and IRF7 were lost in *Ifnar1^−/−^* cells (Fig. 4G). These data indicate a strong dependence of *C. parapsilosis*–induced IL-27 production on IFN-β.

As we have shown that *C. parapsilosis* induced IL-27 via IFN-β, we next sought to determine whether IFN-β could synergize with *C. albicans* to induce IL-27. We observed that low levels of IFN-β increased the production of IL-27 in response to *C. parapsilosis*; however, IFN-β did not synergize with *C. albicans* or heat-killed *C. parapsilosis* (Fig. 4H). In addition to *C. albicans* failing to induce IFN-β production (Fig. 3F), we next asked whether *C. albicans* could actively suppress IL-27 production. To this end, *C. albicans* and *C. parapsilosis* were added to BMDMs at the given ratios. We demonstrated that addition of *C. albicans* actively blocked IL-27 production in response to *C. parapsilosis* (Fig. 4I). In addition, we found that supernatants from BMDMs stimulated with *C. albicans* also blocked *C. parapsilosis*–induced IL-27 production (Fig. 4J), indicating that although *C. parapsilosis* induces IL-27 via IFN-β production, *C. albicans* inhibits IL-27 production via a soluble mediator. As *C. albicans* induces more IL-10 than *C. parapsilosis* (Fig. 3B), we further determined whether IL-10 was responsible for blocking *C. parapsilosis*–induced IL-27. Notably, *C. albicans* continued to block *C. parapsilosis*–induced IL-27 production in the absence of IL-10 (Fig. 4K), indicating the involvement of an as yet unidentified soluble mediator.

### Il27ra^−/−^ mice display enhanced clearance of *C. parapsilosis*

**FIGURE 5.**
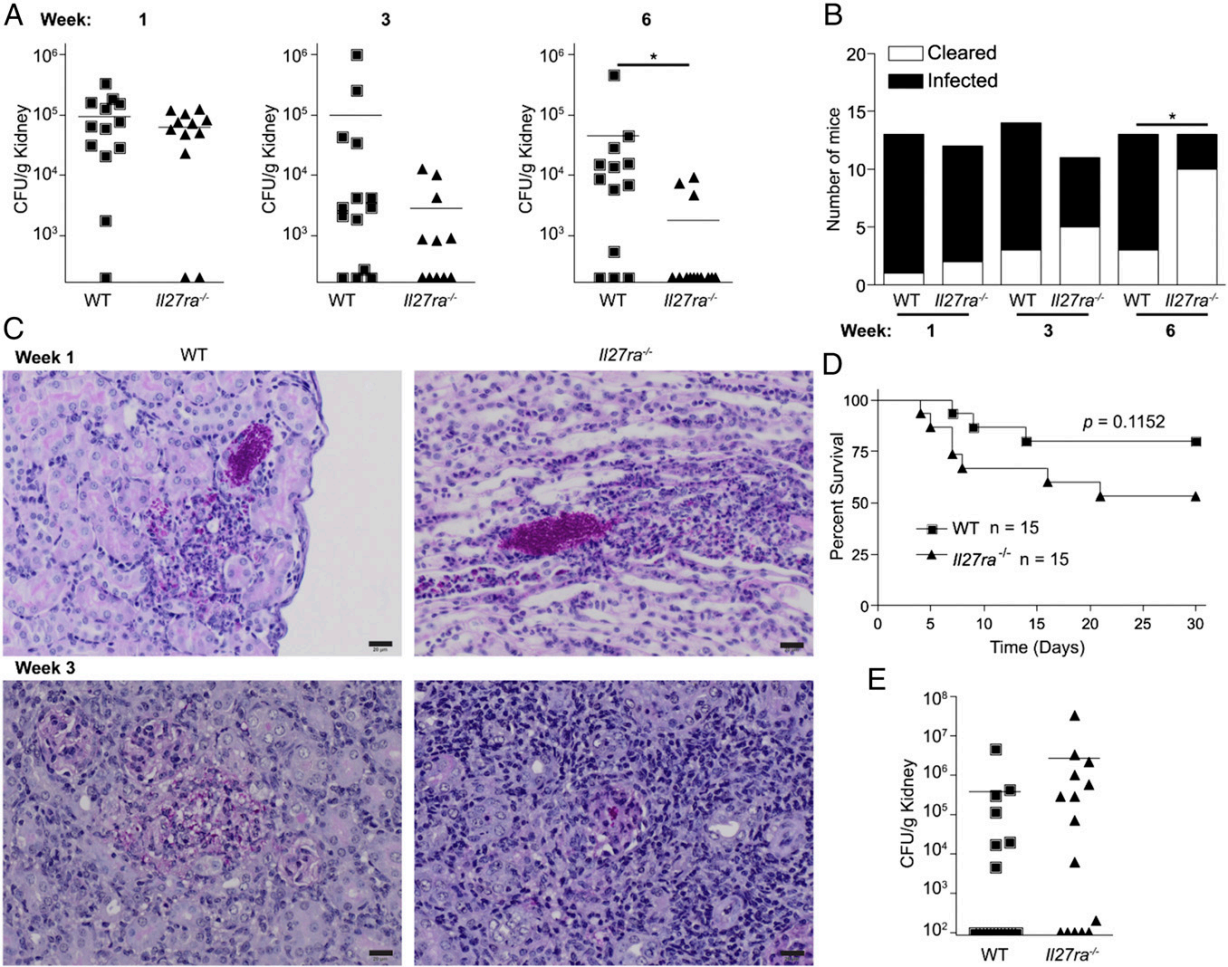
*Il27ra^−/−^* mice display enhanced clearance of *C. parapsilosis*. (**A**) CFU in the kidneys of WT (■) and *Il27ra^−/−^*(▲) mice 1, 3, and 6 wk after i.v. infection with 1.5 × 10^7^ CFU *C. parapsilosis.* Graphs are the cumulative result of two independent experiments. *p < 0.05 (Student *t* test on transformed data). Each symbol represents an individual mouse. (**B**) Number of mice that have cleared the infection (<200 CFU/g kidney) (white bar) or remain infected (black bar) 1, 3, or 6 wk after i.v. infection with 1.5 × 10^7^ CFU *C. parapsilosis.* *p < 0.05 (Student *t* test on transformed data). (**C**) Fungal growth in representative WT (*left panels* [×20 magnification]) or *Il27ra^−/−^* (*right panels* [×20 magnification]) kidneys 1 and 3 wk after i.v. infection with 1.5 × 10^7^ CFU *C. parapsilosis*. Kidney sections were stained with periodic acid–Schiff. Scale bars, 20 μm. (**D** and **E**) Survival curves (D) and CFU in the kidneys (E) of WT (■) and *Il27ra^−/−^*(▲) mice infected i.v. with 1.5 × 10^5^ CFU *C. albicans* for 30 d. Each symbol represents an individual mouse. Graphs are the cumulative result of two independent experiments. (D) *p = 0.1152* (log-rank test), *n* = 15.

*C. parapsilosis* induced significant levels of IL-27 from myeloid cells in vitro and ex vivo (see Fig. 1). Systemic infection (i.v.) of WT mice with *C. parapsilosis* similarly demonstrated in vivo production of IL-27p28 by F4/80^+^CD11b^+^Ly6c^+^ cells and CD11c^+^MHCII^+^ cells (Supplemental Fig. 2). To determine whether IL-27 was important for host pathogen clearance, we next infected WT and *Il27ra^−/−^* mice with *C. parapsilosis*, and *Il27ra^−/−^* mice, compared with WT mice, displayed enhanced fungal clearance, as shown by the progressive reduction in fungal burden in the kidneys by week 6 (Fig. 5A–C). WT and *Il27ra^−/−^* mice displayed comparable fungal burden in the brains at 1 wk post infection, which was followed by complete clearance by week 3 (data not shown). Although *C. albicans* does not induce IL-27 in vitro, we next examined whether the increased clearance was specific for *C. parapsilosis* or whether *C. albicans* induced a similar effect in vivo. *C. albicans* is considerably more pathogenic than *C. parapsilosis*, and as a result infected mice will succumb to the infection. Although *Il27ra^−/−^* mice displayed improved clearance of *C. parapsilosis* (Fig. 5A–C), they did not show improved clearance of *C. albicans* or improved survival (Fig. 5D, 5E). Overall, these data demonstrate that *C. parapsilosis*–induced IL-27 hinders fungal clearance in a systemic *C. parapsilosis* infection model.

### Inflammatory infiltrates are minimally elevated in Il27ra^−/−^ mice

**FIGURE 6.**
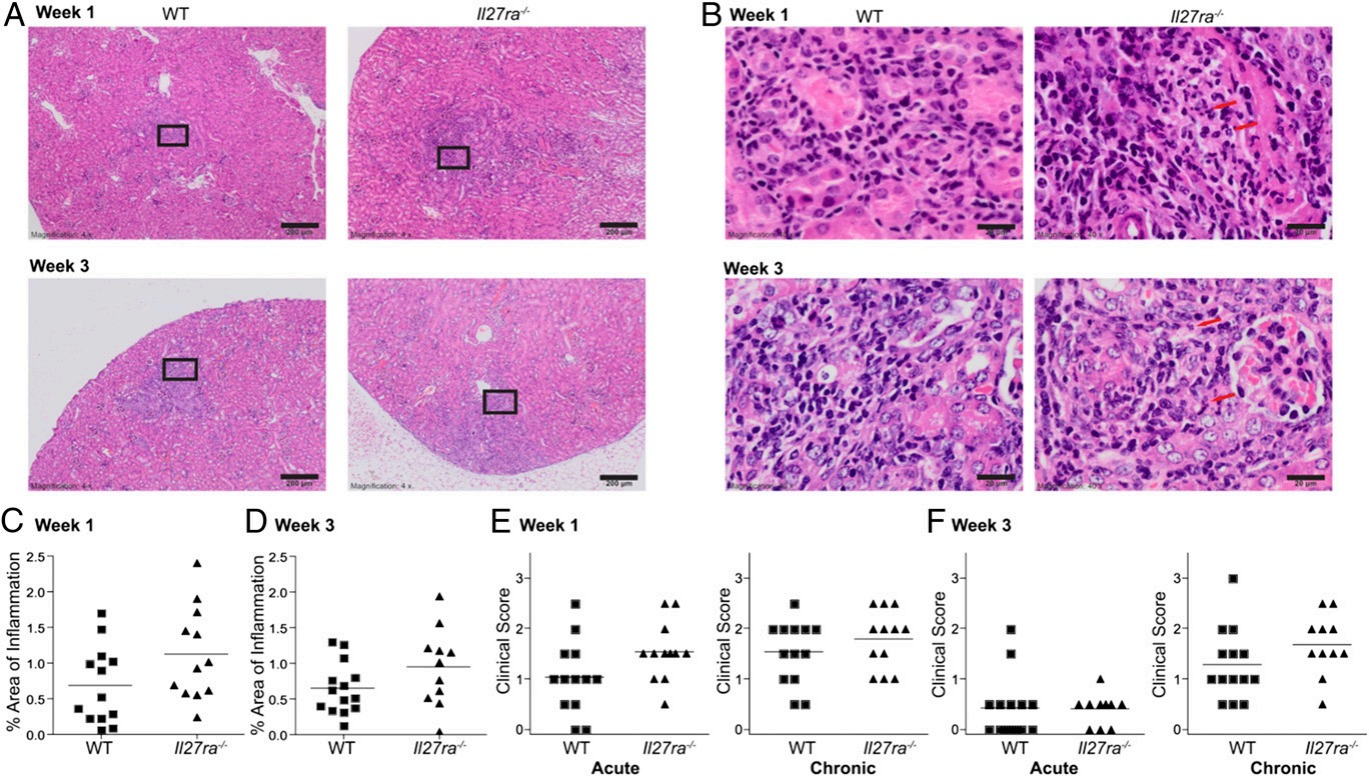
Inflammatory infiltrates are minimally elevated in *Il27ra^−/−^* mice. (**A**) Representative WT (*left panels* [×4 magnification]) or *Il27ra^−/−^* (*right panels* [×4 magnification]) kidneys, 1 and 3 wk after i.v. infection with 1.5 × 10^7^ CFU *C. parapsilosis*. Kidney sections were stained with H&E. Scale bars, 200 μm. (**B**) Higher magnification (×40 magnification) of boxed areas from (A). Scale bars represent 20 μm. Red arrows indicate neutrophil infiltration. (**C** and **D**) Graphs display percentage of area of inflammation in the kidneys from mice 1 wk (C) or 3 wk (D) after i.v. infection with 1.5 × 10^7^ CFU *C. parapsilosis*. ■, WT mice; ▲, *Il27ra^−/−^* mice. (**E** and **F**) Graphs display average acute (neutrophilic) and chronic (lymphocytic) clinical scores from the cortex and medulla of kidneys from mice 1 wk (E) or 3 wk (F) after i.v. infection with 1.5 × 10^7^ CFU *C. parapsilosis* stained with H&E. Graphs are the cumulative result of two independent experiments. Each symbol represents the average score from cortex and medulla for an individual mouse.

Previous studies have demonstrated that *Il27ra^−/−^* mice display increased organ pathological changes in response to various pathogens, including *T. gondii,* malaria-causing parasites (*Plasmodium*), and Leishmania *donovani* (29, 58, 59). Therefore, we next examined whether *Il27ra^−/−^* mice displayed increased inflammatory infiltrates in the kidneys following infection with *C. parapsilosis* (Fig. 6A, 6B). In general, *Il27ra^−/−^* mice displayed a trend toward increased inflammation when compared with WT mice; however, this was not statistically significant (Fig. 6C–F). At 1 wk and 3 wk post infection, the percentage area of inflammation in the kidneys from *Il27ra^−/−^* mice was slightly elevated (Fig. 6C, 6D) compared with that in WT mice. In addition, when compared with WT mice, *Il27ra^−/−^* mice with patchy tubulointerstitial suppurative nephritis showed only slightly elevated foci of neutrophils (acute inflammation) at 1 wk post infection (Fig. 6E) and slightly elevated chronic inflammation (lymphocytes) 3 wk post infection (Fig. 6F). These data indicate that IL-27 delays clearance of *C. parapsilosis* but does not have a significant effect on low-level nephritis caused by *C. parapsilosis.*

### Il27ra^−/−^ mice display increased proinflammatory responses to *C. parapsilosis*

**FIGURE 7.**
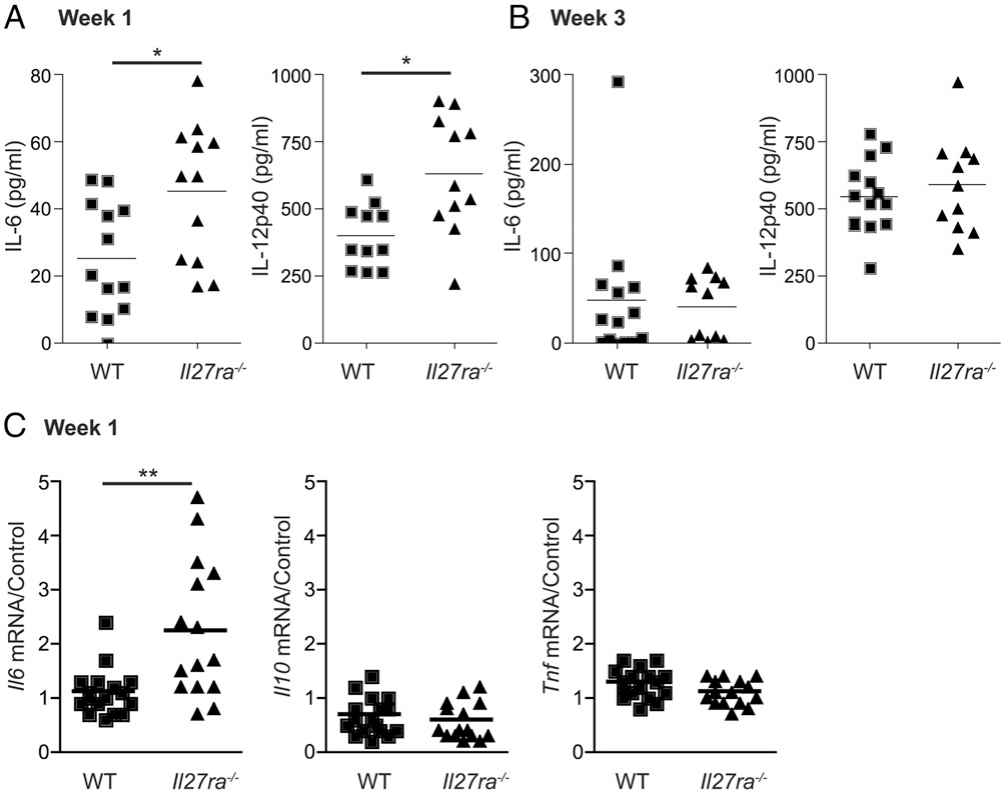
*Il27ra^−/−^* mice display increased proinflammatory responses. (**A** and **B**) Cytokine levels in the serum of WT (■) and *Il27ra^−/−^* (▲) mice 1 wk (A) and 3 wk (B) post infection with 1.5 × 10^7^ CFU *C. parapsilosis.* Graphs are the cumulative result of two independent experiments. *p < 0.05 (Student *t* test). Each symbol represents an individual mouse. (**C**) RNA was isolated from spleen cells 1 wk post infection with 1.5 × 10^7^ CFU *C. parapsilosis*; cDNA was prepared; and *Il6, Il10,* and *Tnf* mRNA transcripts were detected by real-time quantitative PCR. mRNA levels were normalized to *Hprt1*. Graphs are the cumulative result of three independent experiments. Each symbol represents an individual mouse. **p < 0.005 (Student *t* test).

As *Il27ra^−/−^* mice displayed improved clearance of *C. parapsilosis,* we wanted to determine whether IL-27 affects the host inflammatory response to *C. parapsilosis.* Of note, *Il27ra^−/−^* mice demonstrated increased serum levels of some proinflammatory cytokines (IL-12p40, IL-6) 1 wk post infection with *C. parapsilosis* (Fig. 7A). However, by week 3, serum cytokine levels were similar between WT and *Il27ra^−/−^* mice (Fig. 7B). Serum levels of other cytokines, including IL-10 and TNF, were very low or undetected (data not shown). At 1 wk post infection, mRNA levels of *Il6* in the spleen were increased in *Il27ra^−/−^* mice, similar to the serum; however, *Il10* and *Tnf* mRNA levels were similar between WT and *Il27ra^−/−^* mice (Fig. 7C). These data indicate that selective proinflammatory cytokines (IL-12p40, IL-6) are increased in *Il27ra^−/−^* mice at early stages of infection with *C. parapsilosis.*

### Il27ra^−/−^ mice display increased IFN-γ production in response to *C. parapsilosis*

**FIGURE 8.**
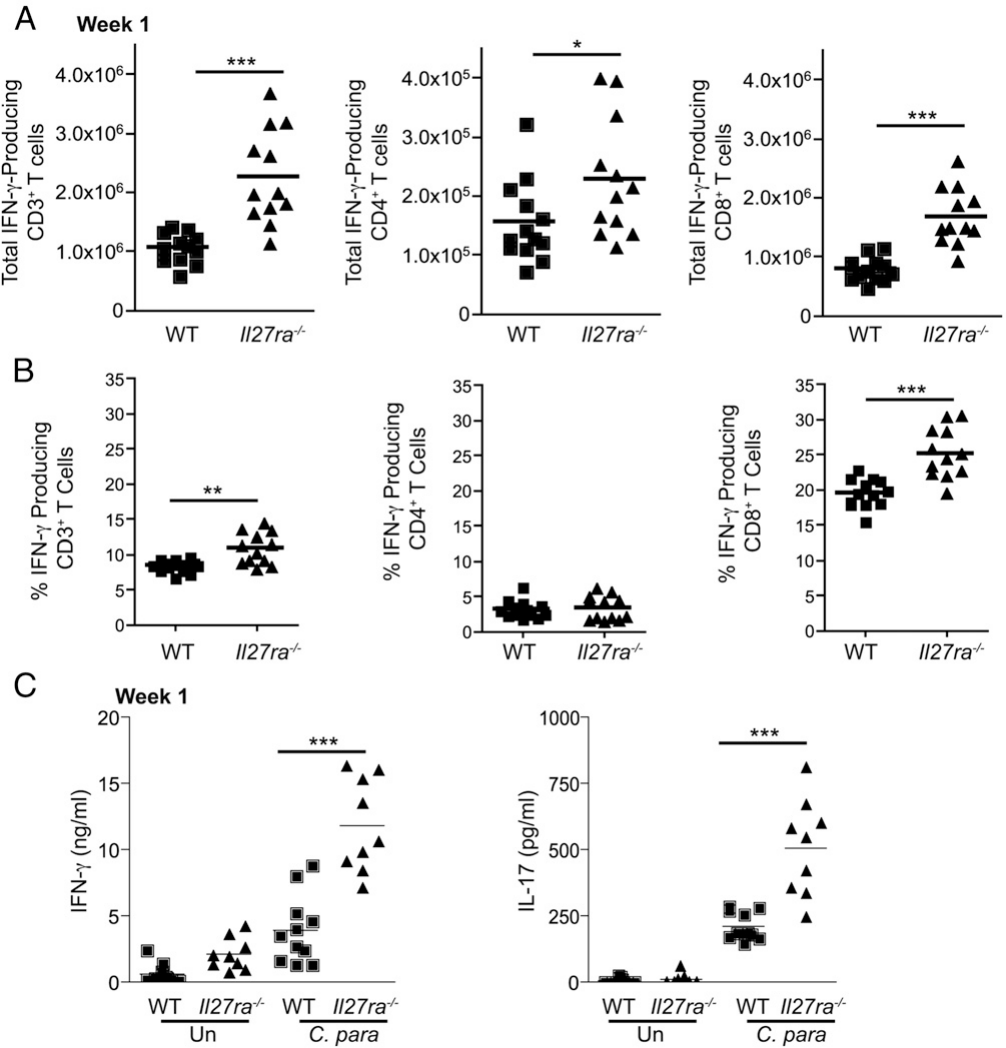
*Il27ra^−/−^* mice display increased IFN-γ production in response to *C. parapsilosis*. (**A** and **B**) WT (■) and *Il27ra^−/−^* (▲) mice were injected i.v. with *C. parapsilosis*. At 1 wk post infection, splenic cells were restimulated with PMA/ionomycin for 4 h. IFN-γ– and IL-17–producing NK1.1^−^CD3^+^ T cells, NK1.1^−^CD3^+^CD4^+^CD8^−^ T cells, and NK1.1^−^CD3^+^CD4^−^CD8^+^ T cells were measured by flow cytometry. Graph displays total number (A) and percentage (B) of CD3^+^NK1.1^−^ T cells, or CD8^+^ or CD4^+^ T cells producing IFN-γ. Each symbol represents an individual mouse. Graphs are the cumulative result of two independent experiments. *p < 0.05, **p < 0.005, ***p < 0.001 (Student *t* test). (**C**) WT and *Il27ra^−/−^* splenic cells 1 wk after i.v. injection of *C. parapsilosis* were left unstimulated or stimulated with *C. parapsilosis* for 48 h. IFN-γ and IL-17 levels in the supernatants were measured by ELISA. Graphs are the cumulative result of two independent experiments. Each symbol represents an individual mouse. ***p < 0.001 (one-way ANOVA, Bonferroni’s posttest).

To examine IFN-γ and IL-17 responses, splenocytes from *C. parapsilosis*–infected mice were restimulated with PMA/ionomycin for 4 h. We observed an increased number (Fig. 8A) and percentage (Fig. 8B, Supplemental Fig. 3) of splenic IFN-γ–producing T cells in *C. parapsilosis*–infected *Il27ra ^−/−^* mice, whereas IL-17–producing cells were very low (Supplemental Fig. 3). Of interest, the majority of these IFN-γ–producing T cells were CD8^+^ rather than CD4^+^ Th cells (Fig. 8A, 8B, Supplemental Fig. 3). In addition, splenocytes from WT and *Il27ra^−/−^* mice 1 wk post infection were restimulated with *C. parapsilosis* for 48 h (Fig. 8C), and IFN-γ and IL-17 levels were analyzed. Cells from *Il27ra^−/−^* mice, compared with cells from WT mice, demonstrated increased robust production of IFN-γ and IL-17 in response to *C. parapsilosis* (Fig. 8C). In contrast, splenic cells from naive mice stimulated with *C. parapsilosis* for 48 h produced little IFN-γ or IL-17 (Supplemental Fig. 3). In addition, we demonstrated that *Foxp3* mRNA levels in the spleen are reduced in *Il27ra^−/−^* mice (Supplemental Fig. 3), suggesting that Treg numbers are reduced in these mice.

Thus, these data indicate that IL-27 inhibits T cell effector cytokine production in response to *C. parapsilosis.*

## Discussion

In this article, for the first time, to our knowledge, we have demonstrated that specific *Candida* spp. induce IL-27 production in myeloid cells and we have identified an important role for IL-27 in the immune response to *C. parapsilosis*. We have shown that *C. parapsilosis*–induced IL-27 production was dependent on TLR7/MyD88 and NOD2 signaling. In addition, IL-27 induction was downstream of IFN-β production, followed by signaling through IFNAR1; STAT1/2 (Supplemental Fig. 4); and the activation of IRF1, IRF3, and IRF7. Similar to findings with other infectious agents, IL-27 inhibits IFN-γ and IL-17 responses (28, 58) and *Il27ra^−/−^* mice demonstrate enhanced clearance of *C. parapsilosis*, compared with WT mice. However, in contrast to findings with other infectious agents, the enhanced IFN-γ responses did not result in severely increased disease in *Il27ra^−/−^* mice. Taken together, these data indicate that blocking IL-27 during *C. parapsilosis* infections could expedite clearance of the pathogen.

On the basis of our data, we believe that various factors control the ability of *C. parapsilosis* to induce IL-27 and the inability of *C. albicans* to induce IL-27. First, we propose that after initial recognition, phagocytosis of *C. parapsilosis* is required to facilitate activation of a pathway involving TLR7 and NOD2 that culminates in the production of IL-27. In agreement with previous findings (52), we show that on a per cell basis, macrophages ingest more *C. parapsilosis* cells than *C. albicans* cells (Fig. 2I, 2J). Our data also indicate that phagocytosed *C. parapsilosis* signals through TLR7 and NOD2. *Candida* RNA has been shown to signal through TLR7 (10, 23), and NOD2 was recently shown to recognize viral RNA (51). Although chitin was also recently shown to signal through NOD2, TLR9, and CARD9 (12), we have demonstrated that *C. parapsilosis*–induced IL-27 is independent of TLR9 and CARD9. Interestingly, TLR9 and NOD2 were shown to colocalize in response to chitin (12). In our studies, we have observed that although some basal TLR7 and NOD2 colocalization occurs, *Candida* spp. induce TLR7 and NOD2 colocalization surrounding the yeast cells, which was particularly robust around *C. parapsilosis* cells. However, further studies are required to determine what signals cause the colocalization of TLR7 and NOD2 and what ligand or ligands signal through TLR7 and NOD2.

Second, we have demonstrated that *C. parapsilosis* induces IFN-β and subsequently IL-27; however, *C. albicans* does not induce IFN-β or the resulting IL-27. *Candida* spp. have recently been shown to induce IFN-β, although the mechanism remains controversial. Bourgeois et al. (10) reported that *C. glabrata* induced significant levels of IFN-β from BMDCs, but not BMDMs, whereas *C. albicans–* and *C. dubliniensis*–induced IFN-β levels from BMDCs were considerably lower. *C. glabrata*–induced IFN-β was produced in a TLR2-, TLR4-, TLR9-, Dectin-1–, and CD11b-independent manner. IFN-β was produced in a phagocytosis-, TLR7/MyD88-, and IRF1-dependent manner, and the resulting IFN-β subsequently signaled through IFNAR1 to induce IRF7 activation, producing a feedback loop resulting in further IFN-β production. Biondo et al. (50) showed that *C. albicans* and *S. cerevisae* induced IFN-β from DCs in a TLR7/TLR9-MyD88– and IRF1/3/7-dependent manner. In contrast to the previous two studies, del Fresno et al. (60) reported that heat-killed *C. albicans* or curdlan-induced IFN-β from DCs was Dectin-1, Dectin-2, Syk, and Card9 dependent. They also showed the Dectin-1–induced IFN-β was IRF5 dependent and IRF3/7 independent. The differences in these studies could be due to the different ligands or species/strains of *Candida* used. In our study, we demonstrate a phagocytosis-, TLR7/MyD88-, IFNAR1-, and STAT1/2-dependent induction of IL-27 downstream of *C. parapsilosis* that is reminiscent of the pathway reported by Bourgeois et al. to induce IFN-β in response to *C. glabrata* (10). Of note, the previous three studies observed either no or very little IFN-β production from BMDMs (10, 50, 60). In contrast, we observed IFN-β production from BMDMs in response to *C. parapsilosis* (Fig. 3F). *C. parapsilosis* was not used in the previous studies, which could explain this discrepancy. In addition, we have observed the dependence of IL-27 production on NOD2. NOD2 has previously been linked to the induction of IFN-β production in response to *Listeria monocytogenes,* which involved synergy with other cytosolic microbial sensors (61). TLR7 and NOD2 are both required for *C. parapsilosis*–induced IL-27, although whether this involves synergy between these two receptors remains to be determined.

Third, we found that in addition to not inducing IL-27, *C. albicans* actively blocks *C. parapsilosis*–induced IL-27 production via a soluble mediator. *C. albicans* is much more virulent than *C. parapsilosis*, and mice infected with *C. albicans* display increased organ pathological changes compared with mice infected with *C. parapsilosis* (data not shown). As IL-27 limits host disease (28, 29, 31, 32), it is possible that the ability of *C. albicans* to block IL-27 production may contribute to the increased pathological changes observed in mice infected with *C. albicans* versus those infected with *C. parapsilosis*. In addition, although we have not investigated this further, it is possible that differences in the intracellular fates of *C. albicans* and *C. parapsilosis* could affect their ability to induce IL-27. As mentioned previously, *C. parapsilosis* is less pathogenic than *C. albicans* (4). Although various *Candida* spp. can promote intracellular survival through modulation of phagosome maturation (62, 63), *C. albicans* can also escape from the phagosome and cause host cell lysis (63–65). It is possible that the increased length of time spent by *C. parapsilosis* in the phagosome through modulation of phagosome maturation (52) may promote sustained activation compared with that in *C. albicans*. Taken together, these data suggest that following phagocytosis, *C. parapsilosis* signals through TLR7 and NOD2, resulting in IFN-β production, which subsequently leads to IL-27 production.

The role of the IL-27R during infectious diseases is complex and has been the subject of numerous investigations in recent years. IL-27R was reported to be critical for resistance to *Trypanosoma cruzi* and *Leishmania major* infections in mice (48, 66); however, other studies reported enhanced clearance of infections and subsequent development of lethal disease in *Il27ra^−/−^* mice, which highlights the complex roles of IL-27 during infection. During the *L. major* infection model *Il27ra^−/−^* mice displayed increased Th2 cell cytokines and reduced Th1 cell responses, whereas during the *T. cruzi* infection model *Il27ra^−/−^* mice displayed increased Th2 and Th1 cytokines (48, 66). The elevated Th2 cell response to *T. cruzi* in *Il27ra^−/−^* mice was responsible for prolonged parasitemia in these mice, and the liver disease in *Il27ra^−/−^* mice was due to the enhanced Th1 cell responses (66). Of interest, reduced bacterial loads were reported in organs of *Il27ra^−/−^* mice infected with *M. tuberculosis* when compared with organs in WT mice. This finding was accompanied by increased production of proinflammatory cytokines (TNF and IL-12p40), CD4^+^ T cell activation, IFN-γ production, and accelerated death due to chronic hyperinflammation (28). In a more recent study, Findlay et al. (58) demonstrated enhanced parasite clearance in a model of *Plasmodium berghei* infection associated with elevated accumulation of IFN-γ–producing CD4^+^ T cells. *Il27ra^−/−^* mice developed severe liver disease that was prevented by depleting CD4^+^ T cells, but not CD8^+^ T cells. This group recently showed that IL-27R signaling inhibits the generation of terminally differentiated KLRG-1^+^ Th1 cells, thereby limiting IFN-γ production from T cells (67). Similarly, during a model of *T. gondii* infection, *Il27ra^−/−^* mice displayed enhanced Th1 cell responses and they developed a lethal inflammatory disease that was rescued by the depletion of CD4^+^ T cells, but not CD8^+^ T cells (29). This group also demonstrated that IL-27 is important for the development of specialized Tregs that control Th1 cell responses at local sites of inflammation (37). In our study, we demonstrated enhanced clearance of *C. parapsilosis* in *Il27ra^−/−^* mice that is associated with increased production of proinflammatory cytokines in the serum and increased IFN-γ and IL-17 production in the spleens. The adaptive T cell response to *C. parapsilosis* has not been widely characterized to date; however, one study in PBMCs demonstrated that *C. parapsilosis* induces both Th1 and Th17 responses (68). Our results clearly demonstrate that *Il27ra^−/−^* mice display an enhanced inflammatory response, which promotes *C. parapsilosis* eradication. Although we have observed enhanced inflammatory responses and slightly elevated inflammation in the kidneys, these changes have not resulted in severe or lethal disease. This may be because *C. parapsilosis* is mainly nonpathogenic in healthy individuals and because in our model it induces a CD8^+^ T cell–biased response with a much lower CD4^+^ T cell response.

Taken together, our results identify a previously unrecognized role for IL-27 in the regulation of *C. parapsilosis* infections. This is the first description of IL-27 production in response to any *Candida* spp. alone. IL-27 is produced through a complex phagocytosis, TLR7/NOD2, IFN-β, IFNAR1-STAT1/2 pathway (Supplemental Fig. 4). The absence of IL-27 signaling promotes enhanced IFN-γ and IL-17 responses that correlate with enhanced clearance of the pathogen. Therefore, blockade of IL-27 signaling during *C. parapsilosis* infections could be considered as a potential therapy; however, further studies are required to determine whether this would be beneficial.

## Supplementary Material

Data Supplement
