# Supplementary material for: IL-27 Induced by Select Candida spp. via TLR7/NOD2 Signaling and IFN-β Production Inhibits Fungal Clearance
Source: J Immunol. 2016 Jun 3;197(1):208–21. doi: 10.4049/jimmunol.1501204 (PMC4911616; doi:10.4049/jimmunol.1501204)
Supplement: Data Supplement [file JI_1501204.zip › JI_1501204_Supplemental_Figures_1.pdf]

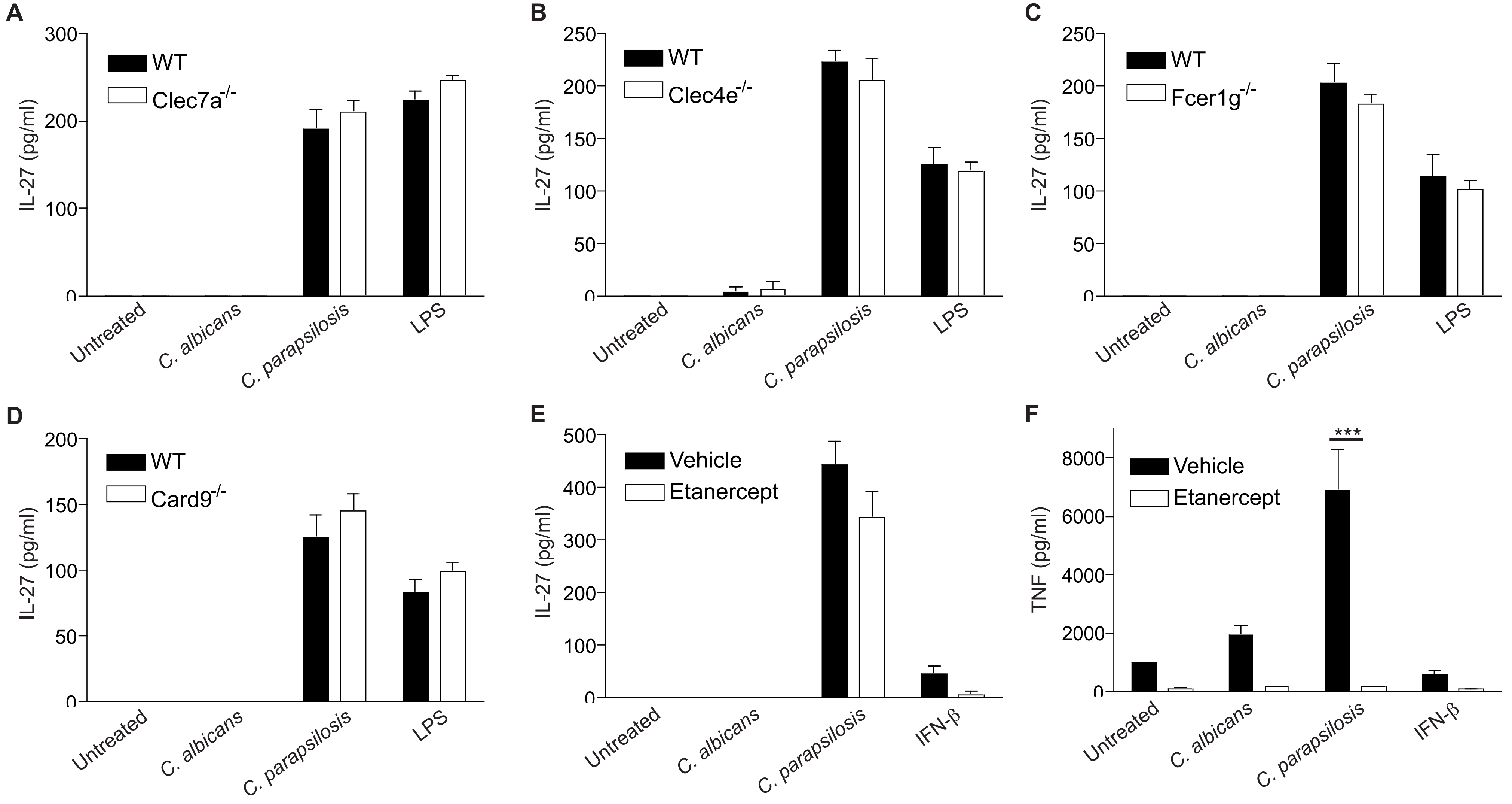

Figure S1. *Candida*-Induced IL-27 Production is Dectin-1, Mincle, FcεRIγ and CARD9-independent with Minor Dependence on TNF. (A-D) BMDMs from WT and *Clec7a*$^{-/-}$ mice (A), WT and *Clec4e*$^{-/-}$ mice (B), WT and *Fcer1g*$^{-/-}$ mice (C) and WT and *Card9*$^{-/-}$ mice (D) were stimulated with *Candida* spp. Cytokine levels in the supernatants were measured after 24h incubation. (E-F) BMDMs from WT mice were stimulated with *Candida* spp. in the presence of Vehicle control or 25μg/ml Etanercept. Cytokine levels in the supernatants were measured after 24h incubation. For all graphical data, results are presented as means +/- s.e.m. of three replicates and data are representative of 2-3 independent experiments.

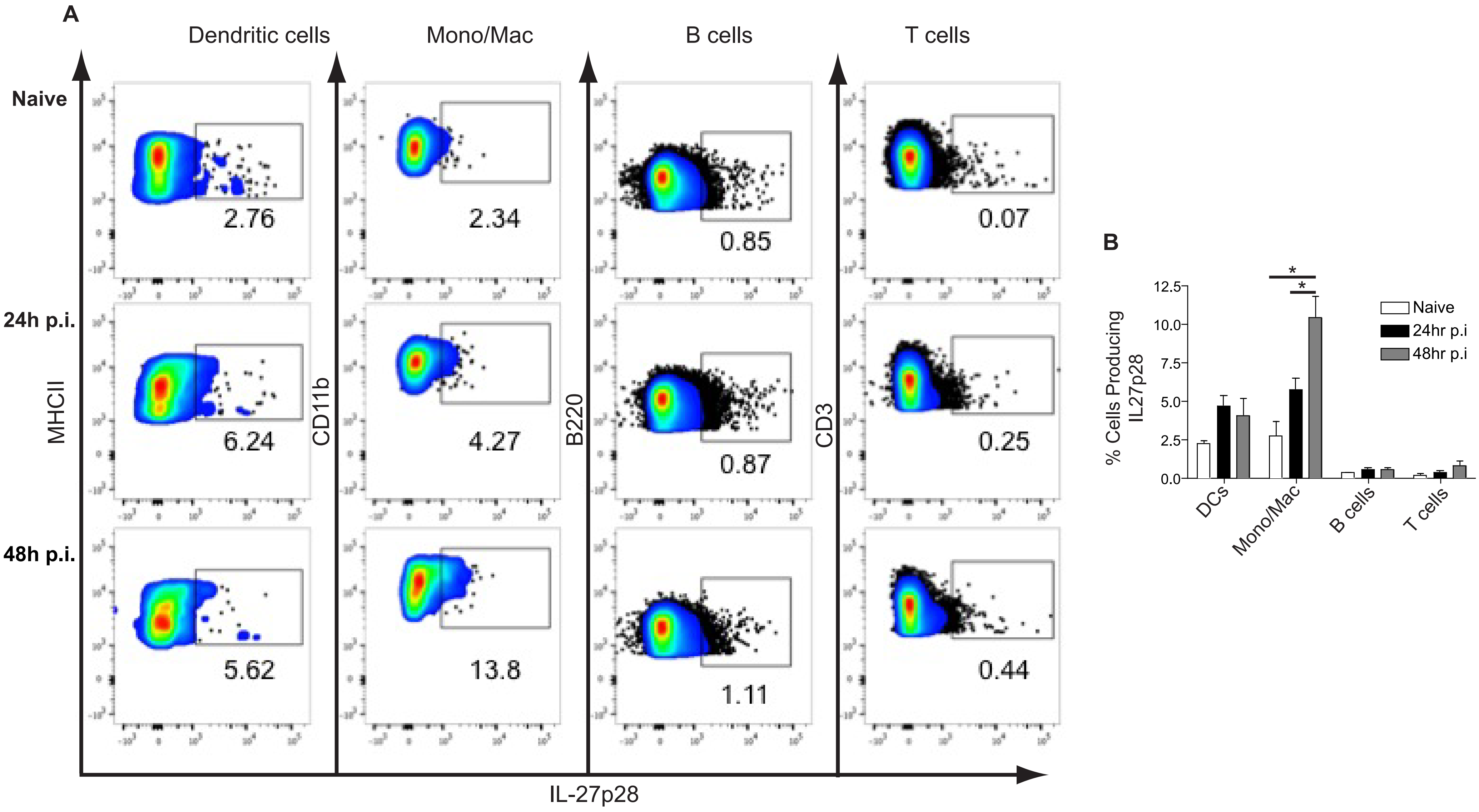

Figure S2. IL27 is Produced by Myeloid Cells Following Infection with *C. parapsilosis*.
(A-B) WT mice were injected intravenously with *C. parapsilosis*. IL-27p28 producing splenic $CD11c^{+}MHCII^{+}$ DCs, $F4/80^{+}CD11b^{+}Ly6c^{+}$ monocytes/macrophages, $B220^{+}$ B cells and $CD3^{+}$ T cells were measured by flow cytometry from naive mice or 24-48h post infection (p.i.). Flow plots are representative of 3-4 mice per group and data are representative of 2 independent experiments. (B) Graph displays mean +/- s.e.m. % cells expressing IL-27p28 from 3-4 mice analyzed by flow cytometry. Graph is representative of 2 independent experiments.*$p<0.05$ (1-way ANOVA, Bonferroni's post-test).

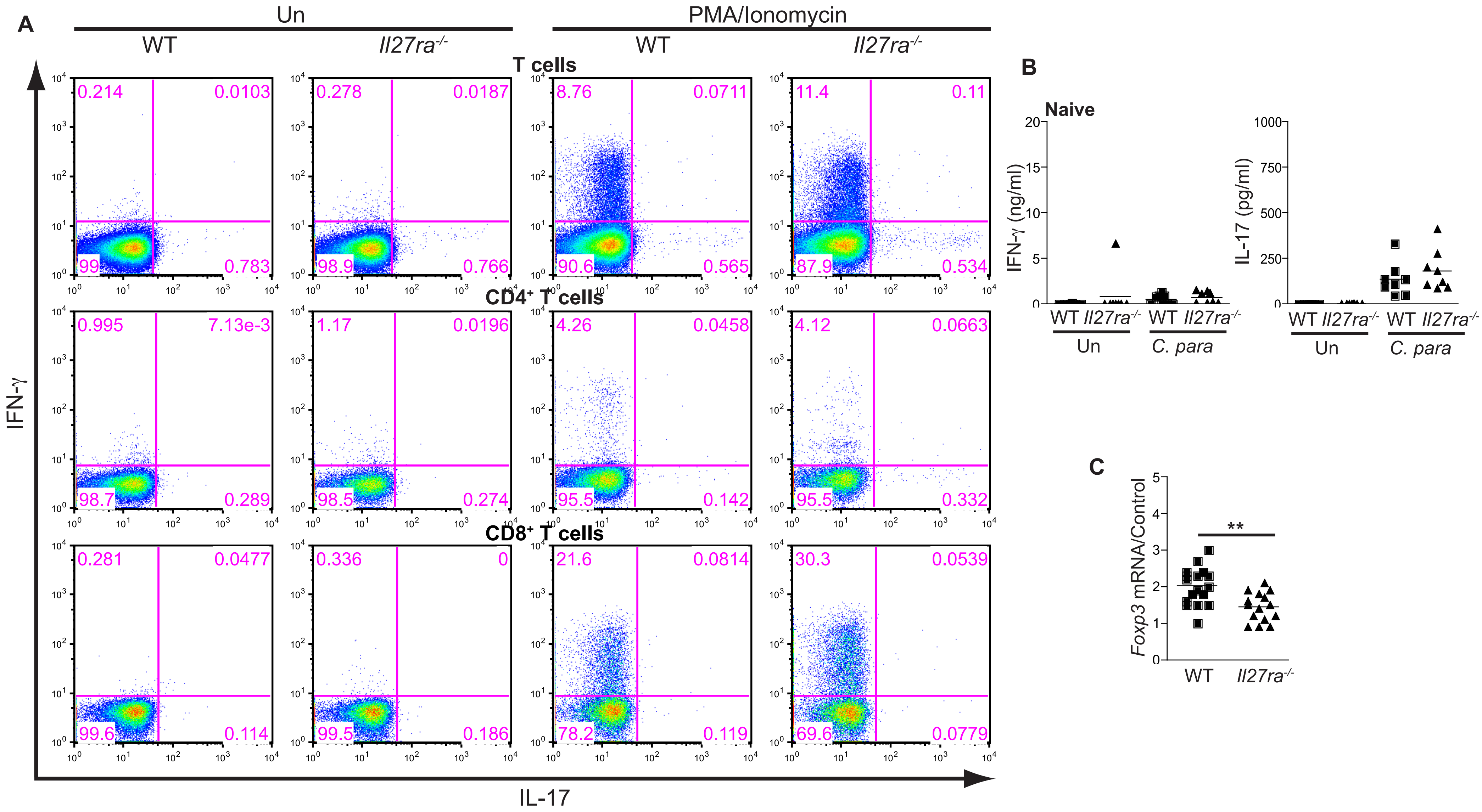

Figure S3. *Il27ra$^{-/-}$* mice display enhanced IFN-γ response to *C. parapsilosis*.
(A) WT and *Il27ra$^{-/-}$* mice were injected intravenously with *C. parapsilosis*. One week after infection splenic cells were left unstimulated or re-stimulated with PMA/Ionomycin for 4h. IFN-γ and IL-17 producing NK1.1$^-$CD3$^+$ T cells, NK1.1$^-$CD3$^+$CD4$^+$CD8$^-$ T cells and NK1.1$^-$CD3$^+$CD4$^-$CD8$^+$ T cells were measured by flow cytometry. Flow plots are representative of 5-6 mice per group and data are representative of 2 independent experiments. (B) WT and *Il27ra$^{-/-}$* splenic cells from naïve mice were left unstimulated or stimulated with *C. parapsilosis* for 48h. IFN-γ and IL-17 levels in the supernatants were measured by ELISA. Graphs are the cumulative result of 2 independent experiments. Each symbol represents an individual mouse. (C) WT and *Il27ra$^{-/-}$* mice were injected intravenously with *C. parapsilosis*. One week after infection splenic cells were processed, RNA was isolated, cDNA was prepared and *Foxp3* mRNA transcripts were detected by real-time qPCR. mRNA levels were normalized to Hprt1. Each symbol represents an individual mouse. *p<0.05 **p<0.005 (Student's *t* test)

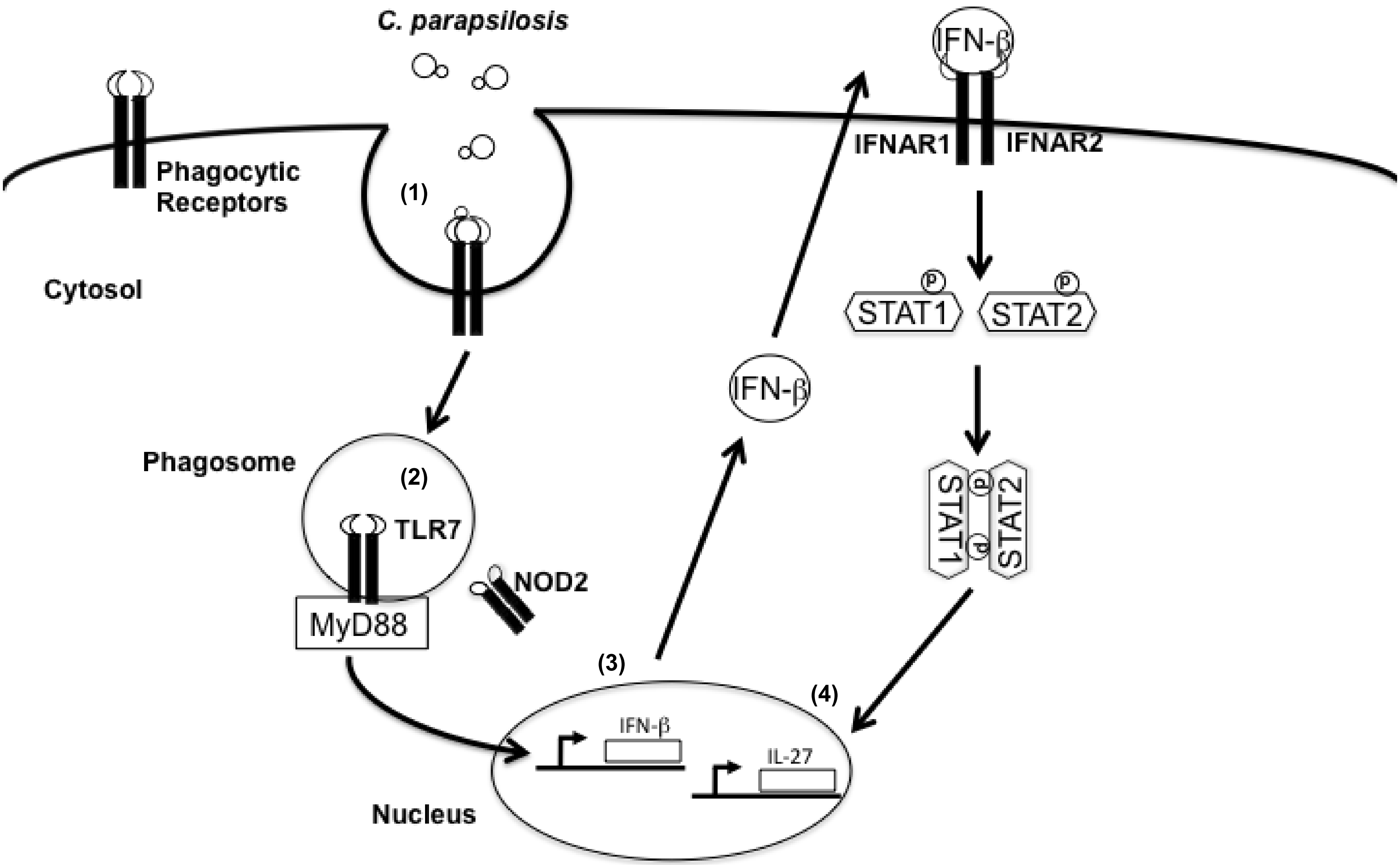

Figure S4. Model of *C. parapsilosis* induction of IL-27.
(1) *C. parapsilosis* bind to cell surface receptors on BMDM and is phagocytosed. (2) TLR7/MyD88 and NOD2 signaling are activated, resulting in the production of IFN-β. (3) IFN-β signals through the IFNAR1/2-STAT1/2 pathway and (4) induces IL-27 production.
